# Glacial Lake Expansion in the Central Himalayas by Landsat Images, 1990–2010

**DOI:** 10.1371/journal.pone.0083973

**Published:** 2013-12-20

**Authors:** Yong Nie, Qiao Liu, Shiyin Liu

**Affiliations:** 1 Institute of Mountain Hazards and Environment, Chinese Academy of Sciences, Chengdu, China; 2 State Key Laboratory of Cryosphere Sciences, Cold and Arid Regions Environmental and Engineering Research Institute, Chinese Academy of Sciences, Lanzhou, China; NASA Jet Propulsion Laboratory, United States of America

## Abstract

Glacial lake outburst flood (GLOF) is a serious hazard in high, mountainous regions. In the Himalayas, catastrophic risks of GLOFs have increased in recent years because most Himalayan glaciers have experienced remarkable downwasting under a warming climate. However, current knowledge about the distribution and recent changes in glacial lakes within the central Himalaya mountain range is still limited. Here, we conducted a systematic investigation of the glacial lakes within the entire central Himalaya range by using an object-oriented image processing method based on the Landsat Thematic Mapper (TM) or Enhanced Thematic Mapper (ETM) images from 1990 to 2010. We extracted the lake boundaries for four time points (1990, 2000, 2005 and 2010) and used a time series inspection method combined with a consistent spatial resolution of Landsat images that consistently revealed lake expansion. Our results show that the glacial lakes expanded rapidly by 17.11% from 1990 to 2010. The pre-existing, larger glacial lakes, rather than the newly formed lakes, contributed most to the areal expansion. The greatest expansions occurred at the altitudinal zones between 4800 m and 5600 m at the north side of the main Himalayan range and between 4500 m and 5600 m at the south side, respectively. Based on the expansion rate, area and type of glacial lakes, we identified 67 rapidly expanding glacial lakes in the central Himalayan region that need to be closely monitored in the future. The warming and increasing amounts of light-absorbing constituents of snow and ice could have accelerated the melting that directly affected the glacial lake expansion. Across the main central Himalayas, glacial lakes at the north side show more remarkable expansion than those at the south side. An effective monitoring and warning system for critical glacial lakes is urgently needed.

## Introduction

Glacial lake outburst floods (GLOFs) pose a serious hazard in many high mountain communities around the world [1] and have increasingly been given attention in recent years due to the associated catastrophic damages and fatalities [2]–[7]. The Himalayan mountainous area is one of the hardest hit regions by GLOFs [2], [4], [7]–[10]. GLOFs, or glacial lake expansions, can also be seen as an indirect indicator of climate change. Consistent with the expectations of global warming, most Himalayan glaciers are losing mass [5], [11], [12]. Glacial recession in the Himalayas has resulted in the development of glacial lakes, especially moraine-dammed glacial lakes [12], which have increased the potential risk for GLOFs in this region.

Remote sensing has been proven to be the most useful method for the monitoring and early detection of GLOF hazards in remote mountainous regions [1], [3], especially as it offers the capability to investigate a large area. Investigating glacial lakes over a large area and monitoring glacial lake dynamics (temporal and spatial changes) are of high priority for the mitigation of GLOF hazards. Glacial lake inventories and change assessments were reported for the Nepal [3], [4], [6], [9], Chinese [13], Indian [8] and Bhutan Himalayas [14] based on remote sensing, whereas a full regional inventory for the entire Himalaya region has not been undertaken due to political constraints. Although a regional assessment of glacial lake distribution and evolution in the Hindu Kush Himalayas had been reported [15], these studies are still limited to a few typical regions.

**Figure 1:**
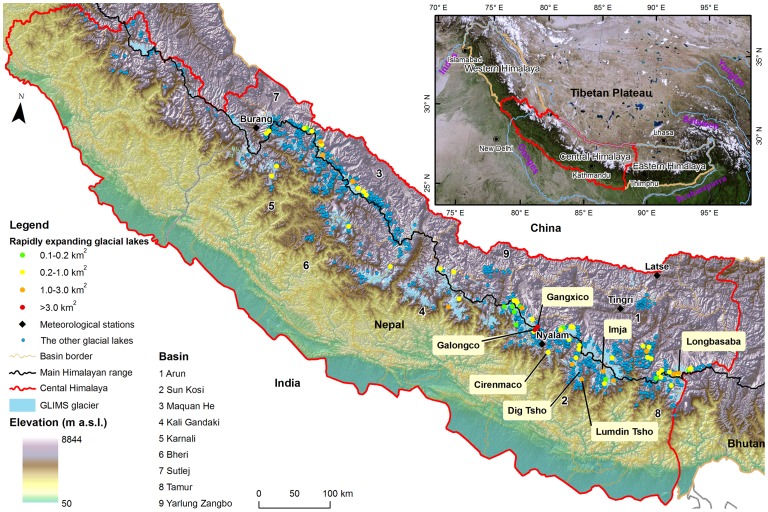
Distribution of glacial lakes in the central Himalayas.

Most GLOF events documented in the Himalayas occurred in the central Himalayas and typically consisted of outburst floods from moraine-dammed glacial lakes [2], [4], [7], [9], [10]. The central Himalayas have an area of approximately 28×10^4^ km^2^ [12], forming a natural boundary between China and Nepal, as well as between China and India (Figure 1). Documented GLOF events (such as Lake Cirenmaco and Dig Tsho) in the central Himalayas destroyed a downstream hydropower station, road and bridge, killed hundreds of people and caused millions of dollars in economic losses [2], [7], [9], [16]. Potentially dangerous glacial lakes in the central Himalayas, such as Lake Imja [6], [9], Ludin Tsho [9], Gangxico, Galongco [2], [17] and Lake Longbasaba [7], have been identified by many scientific researchers.

This study focuses on the entire central Himalaya region, aiming to analyze the glacial lake changes across the entire region from 1990 to 2010, spanning four time points (1990, 2000, 2005 and 2010), and to identify the rapidly expanding glacial lakes that need to be closely monitored in the future. Further discussions will be made on the discrepancy between the spatial distribution and changes in these glacial lakes between the north and south sides of the main Himalayan range. This study significantly expands on previous work by using homogeneous satellite imagery to investigate the glacial lakes across the entire central Himalayan mountain range. This research will promote the awareness of the hazard potential of glacier lakes by providing some fundamental information for the future assessment of disaster mitigation.

## Data and Methods

The precisely ortho-rectified images of Landsat satellites with a spatial resolution of 30 m were used in this study. A total of 56 Landsat TM/ETM images were downloaded from the Global Land Survey dataset (Global Land Survey (GLS) 1990, GLS2000, GLS2005 and GLS2010) of the United States Geological Survey (USGS) and the Global Land Cover Facility (GLCF). The probability of cloud cover in mountainous areas is high [18]–[20], which makes it particularly difficult to acquire same-month images for glacial lake monitoring over the whole study area. In order to reduce the effect of the difference in acquisition times, high-quality images available from similar seasons were selected, focusing on autumn and winter. The months in which the Landsat images used in this study were acquired include September (8 scenes), October (20 scenes), November (20 scenes), December (6 scenes) and February (2 scenes). A hydrological observation at Rongbuk Glacier, Mount Qomolangma (Everest), central Himalayas revealed an ablation period of glaciers mainly from June to August, and river discharge declined gradually, ending after September [21]. Areal variation of glacial lakes in a high-altitudinal cold environment from September to February was slight; thus, the effect of differences in the acquisition time on the glacial lake change should be negligible. Although the quality of the image strips in Landsat-7 ETM after 2003 are reduced, the glacial lake extents can be clearly distinguished from the improved ETM images through the malfunction of the scan lines corrector (SLC-off) repair method and geo-rectification [22].

Compared with traditional automatic mapping methods, such as supervised classification [23] or decision tree [24], object-oriented image processing is an advanced method widely used as an automatic feature extraction technology due to higher accuracy and efficiency for classification [20], [22], [25]. The Normalized Difference Water Index (NDWI) [1], [26] and Normalized Difference Snow/Ice Index (NDSII) [27] were used to derive individual glacial lakes or glacier extent. We developed an improved method that combines the object-oriented image processing method and expert knowledge using the two indices to extract a preliminary glacial lake extent. The minimum area for lake mapping from TM or ETM is 0.0081 km^2^, or 9 pixels. The workflow included NDWI and NDSII calculation, band stacking, application of edge-based segmentation algorithms [28], definition of classification rules and exporting the extracted output. The detailed workflow and procedure for the extraction of glacial lakes were reported in our previous studies [19], [20], [22].
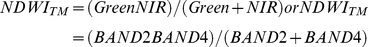
(1)

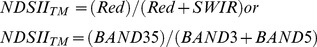
(2)


After the automatic extraction of glacial lake features, visual inspection and correction based upon the field experience on glacial lakes and Google Earth images must be employed to eliminate the misinterpretation of lakes due to shadow, lake ice formation, cloud and snow cover features across the four time points. The missing parts of the lake boundaries from the ETM striping were manually corrected based on earlier TM or ETM images without striping. Centroids of shapes were calculated as the index of label points for each glacial lake. Lakes with independent labels were then joined spatially into the areal attributes for lakes across the four time points. Cross-validation and modification for each glacial lake was conducted according to the time series areal variation. Accuracy assessment of the classification is difficult [15], because the field measurements of most glacial lake outlines are impossible due to their steep edges in high and cold mountainous areas. However, field experiences still help in confirming the locations and shapes of glacial lakes during the process of visual correction. A total of 131 glacial lakes in 2010 were randomly selected for accuracy assessment. These lake outlines were converted into. KMZ format and overlaid with Google Earth images. The results indicated that approximately 98% of glacial lake outlines were well matched in high resolution images by eliminating the difference in acquisition year and spatial resolution scale. The same selected 131 glacial lakes were also cross-checked across the four time points, and there were no clear errors. Therefore, a high-quality final glacial lake dataset was established. Generally, the uncertainty in the measurement of the glacial lake area was estimated by assuming an error of ± 0.5 or ± 1 pixel [6], [15], [29]. In this study, because we used Landsat images with the same resolution of 30 m for the overall mapping and the co-registration errors were less than 1 pixel, we used ± 30 m (1 pixel) as the co-registration error. The formula of uncertainty in the area calculation reported by Ye *et al.* [30] was adopted.

The GLIMS glacier inventory data (available: http://glims.org/download/accessed 2013 Nov 20.) [31] was used to view the spatial relationships between glaciers and lakes. Misinterpretations of the outlines of glaciers determined by automated classification of satellite images were corrected by visual inspection and modification. Based on the locations of the glacial lakes, they are classified into (1) pro-glacial lakes that make contact with the glaciers, (2) pro-glacial lakes without ice contact and (3) supra-glacial lakes, following the method suggested by Gardelle *et al* [15].

In this study, the identification of rapidly expanding glacial lakes was based on the observations that (1) a lake area larger than 0.1 km^2^ is within a threshold size of potentially dangerous lakes [7]; (2) lake areal expansion is more than 20% [32] from 1990 to 2010; (3) a glacial lake is in contact with modern glaciers that are supplied directly by glacial melt water [7], [15].

**Figure 2:**
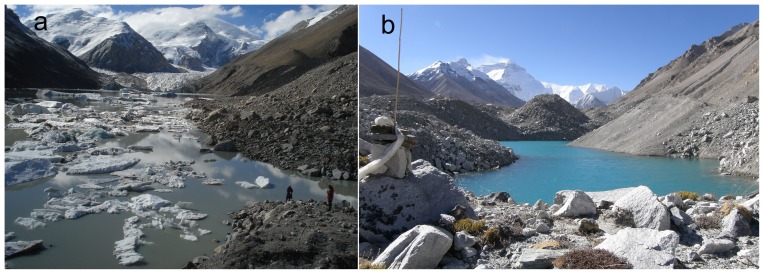
Field observations for glacial lakes: (a) the rapidly expanding Lake Longbasaba in 2012; (b) an areally increasing glacial lake at the Middle Rongbu Glacier near Mount Qomolangma (Everest) in 2008.

Other data used in this study include SRTM DEM [33]; a field survey on glacial lakes in 2008, 2009 and 2012 by our work team in the Chinese Himalayas (Figure 2); weather observations at four meteorological stations (Tingri, Nyalam, Latse and Burang) from the China Meteorological Administration; and digitalized Himalayan mountain range extent results based on Bolch *et al* [12].

## Results

### 3.1 The distribution and changes of glacial lakes

Glacial lakes in the central Himalaya showed obvious expansion in area and increase in number from 1990 through 2010. In total, there were 1,314 glacial lakes (197.22±0.004 km^2^) in 2010 within the study area. The total area of glacial lakes expanded by 28.81±0.012 km^2^ (17.11%) from 1990 to 2010. Larger glacial lakes expanded more rapidly than the smaller ones. The total area has increased by 10.73±0.012 km^2^ for lakes with areas greater than 1.0 km^2^ but less than or equal to 3.0 km^2^ and 9.05±0.012 km^2^ for those greater than 3.0 km^2^ (Table 1).

**Table 1:** Number and area of glacial lakes at different areal ranks in 1990, 2000, 2005 and 2010.

Areal range(km^2^)	1990		2000		2005		2010		1990–2010 changes	
	Count	km^2^	Count	km^2^	Count	km^2^	Count	km^2^	Count	km^2^
≤0.1	841	35.55	915	37.95	919	38.06	922	37.41	81	1.86
0.1–0.2	170	23.72	176	24.27	188	26.38	190	26.66	20	2.94
0.2–1.0	161	63.15	176	70.00	170	68.21	171	67.38	10	4.23
1.0–3.0	14	24.07	16	24.18	19	28.17	24	34.81	10	10.73
>3.0	5	21.91	7	28.88	7	30.02	7	30.96	2	9.05
Total	1191	168.40	1290	185.28	1303	190.84	1314	197.22	123	28.81

In 1990, there were 350 glacial lakes with areas greater than 0.1 km^2^. The number increased by 42 from 1990 to 2010. There were 31 glacial lakes larger than 1 km^2^ in 2010, of which 12 lakes were newly formed after 1990. Glacial lakes larger than 1 km^2^ have expanded in area by 43% during the last 20 years.

The process of glacial lake change was very complex, consisting of self-expansion, new formation and the disappearance of glacial lakes. From 1990 to 2010, the number of newly formed glacial lakes was greater than the number that disappeared. More than 80% of lake expansions consisted of the growth of existing glacial lakes over three spans of time (Table 2). Thus, newly formed lakes are not the biggest contribution to lake expansion in the central Himalayas.

**Table 2:** Processes of glacial lake changes in areas and numbers from 1990 to 2010.

Year	Total number	Total change area (km^2^)	Newly formed		Disappeared		Existing		
			Count	km^2^	Count	km^2^	Count	Change (km^2^)	Contribution of the area change (%)
1990	1191	–	–	–	–	–	–	–	–
2000	1290	+16.88	110	3.84	11	0.62	1180	+13.67	80.97
2005	1303	+5.54	22	1.16	9	0.18	1281	+4.57	82.45
2010	1314	+6.39	20	0.85	9	1.61	1294	+7.15	100.00

### 3.2 Difference in changes in glacial lake types

Pro-glacial lakes in contact with glaciers have a higher expansion rate than supra-glacial lakes. Pro-glacial lakes in contact with glaciers increased in area by 23.87 km^2^, which accounted for 82.85% of the total glacial lake expansion from 1990 to 2010. Pro-glacial lakes disconnected from glaciers have the lowest expansion rates (Table 3).

**Table 3:** Changes to pro- and supra-glacial lakes from 1990 to 2010.

Type	Increasing area (km^2^)	Percent (%)
Pro-glacial lakes in contact with glaciers	23.87	82.85
Supra-glacial lakes	3.26	11.30
Pro-glacial lakes disconnected from glaciers	1.68	5.85
Total	28.81	100.00

### 3.3 Altitudinal difference in distribution and change of glacial lakes

**Figure 3:**
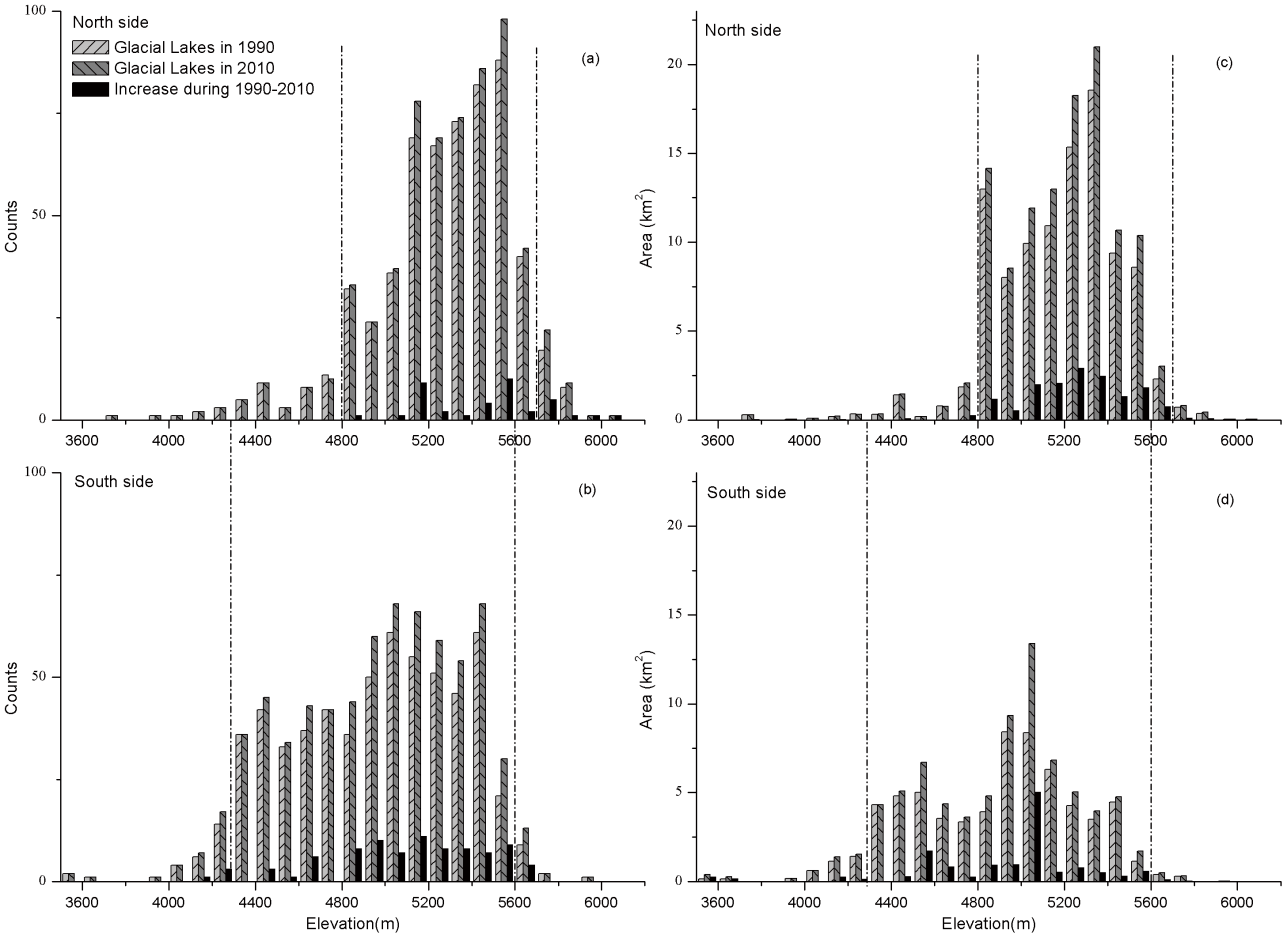
Distribution and change of glacial lakes at different altitudinal zones on the north (a and c) and south sides (b and d) of the main central Himalayan range.

A clear altitudinal distinction in the distributions and changes of glacial lakes was identified at the north and south sides of the main Himalayan range (Figure 3). All the glacial lakes were located between 3500 m and 6100 m a.s.l. with a simple normal distribution. The majority of glacial lakes were distributed in an altitudinal zone from 4800 m to 5700 m at the north side and from 4300 m to 5600 m at the south side, accounting for more than 87% and 93% of all glacial lakes in quantity and area, respectively. The elevations of the glacial lakes where the majority of the expansion was occurring (more than 91%) are between 4800 m and 5600 m a.s.l. at the north side and between 4500 m and 5600 m a.s.l. at the south side from 1990 to 2010. The maximum frequency of occurrence of newly formed glacial lakes was observed between 5100 m and 5200 m a.s.l. at the south side and between 5500 m and 5600 m a.s.l. at the north side. The zone in which the greatest increase in glacial lake area occurred was between 5000 m and 5100 m a.s.l. at the south side.

### 3.4 Comparison of glacial lakes at the north and south sides of the main Himalayan range

The distribution and change of glacial lakes in the central Himalayas is different between the north side and south side of the mountain ridge. On the north side of the main central Himalayan range, the increasing areas for glacial lakes of all types and for rapidly expanding glacial lakes were greater than those at the south side (Table 4). This trend was opposite to the expansion trend in the Bhutan eastern Himalayas [14]. This finding indicates that a regional differentiation in glacial lake changes among the entire Himalayas may exist. In this study, approximately 63% of rapidly expanding glacial lakes was found at the northern side of the main central Himalayan range (Figure 1).

**Table 4:** Contrast of glacial lakes on the north and south sides of the main central Himalayan range.

Location	All glacial lakes			Rapidly expanding glacial lakes	
	Area in 1990 (%)	Area in 2010 (%)	Increasing area 1990–2010 (%)	Count (%)	Increasing area 1990–2010 (%)
North side	60.92	59.84	53.48	62.69	58.23
South side	39.08	40.16	46.52	37.31	41.77

### 3.5 Rapidly expanding glacial lakes

We identified 67 rapidly expanding glacial lakes in the central Himalayas, distributed into 9 river basins (Figure 1 and Table 5). The top three rapidly expanding glacial lakes by quantity were in the Arun (38.81%), Sun Kosi (22.39%) and Maquan He (11.94%) river basins. Rapidly expanding glacial lakes in the Sun Kosi river basin had the greatest expanding area (7.78 km^2^) from 1990 to 2010, implying a higher potential risk for flooding that is cause for greater concern.

**Table 5:** Rapidly expanding glacial lakes in different river basins.

River basin	Count	Percent (%)	1990	2010	1990–2010
			Area (km^2^)	Area (km^2^)	Expanding area (km^2^)
Arun	26	38.81	9.19	16.56	7.37
Sun Kosi	15	22.39	9.36	17.14	7.78
Maquan He	8	11.94	5.12	8.36	3.25
Kali Gandaki	6	8.96	1.18	1.93	0.75
Karnali	4	5.97	0.49	1.40	0.92
Bheri	2	2.99	0.16	0.46	0.30
Sutlej	2	2.99	0.45	0.76	0.31
Tamur	2	2.99	0.15	0.58	0.43
Yarlung Zangbo	2	2.99	0.18	0.50	0.32
Total	67	100.00	26.26	47.69	21.43

## Discussion

### 4.1 Typical glacial lake changes

**Figure 4:**
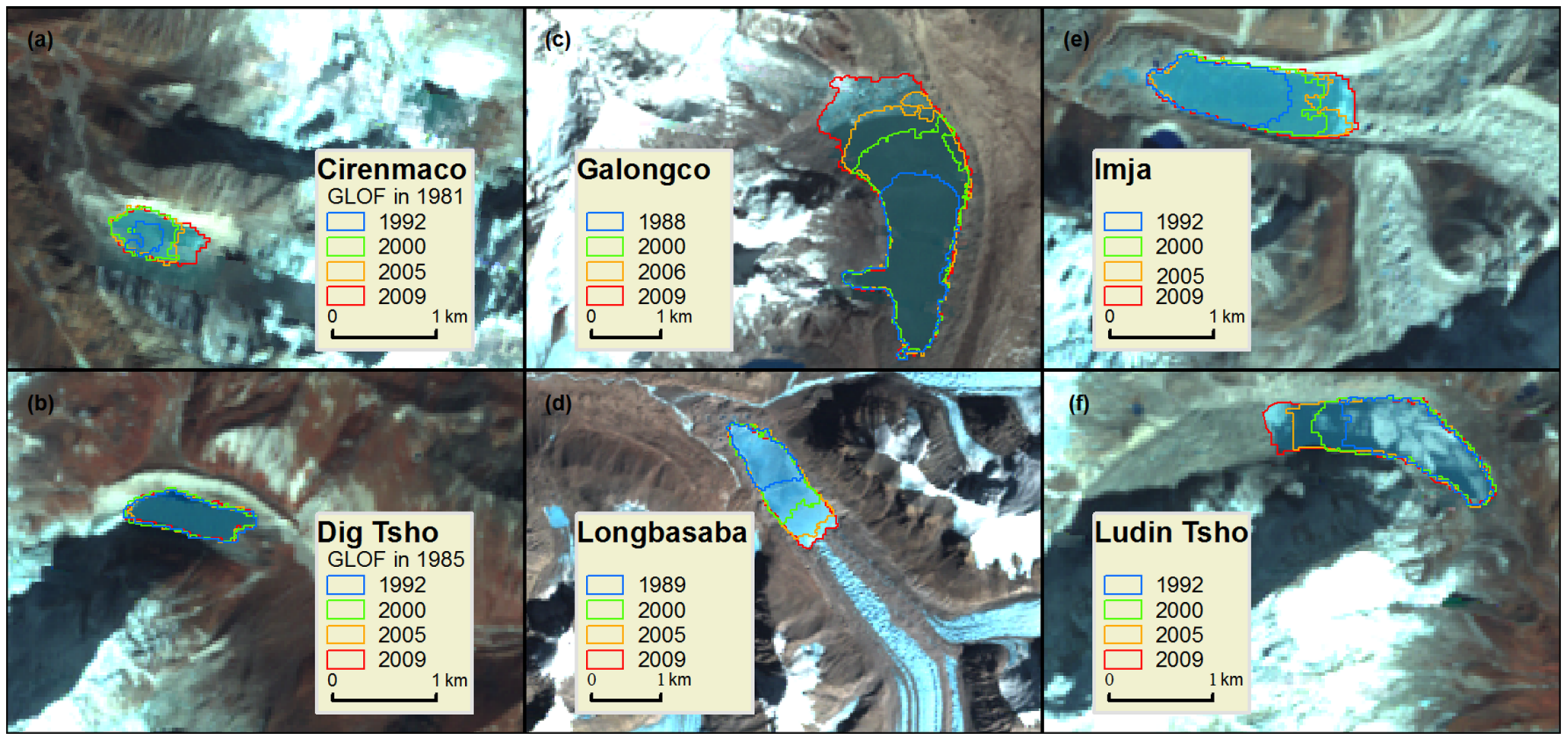
GLOF events and changes in typical critical glacial lakes within the study area.

Most glacial lakes will be safe for several years after a GLOF. For example, the Dig Tsho glacial lake (Figure 4b) burst on 4 August 1985 [9], but it remained stable with almost no changes from 1992 to 2009 after the GLOF. However, some formerly outburst moraine-dammed lakes, for example, Lake Cirenmaco, which breached on July 11, 1981, underwent remarkable expansion between 1992 and 2009 (Figure 4a and Table 6). Based on the criterion that we mentioned above, Cirenmaco was classified as a rapidly expanding glacial lake, indicating a high potential risk.

**Table 6:** List of typical glacial lakes in the central Himalayas.

Glacial lake	Location	Sensors	Period	Area change (%)	Reference	Remarks
Cirenmaco	86.065828E,28.067165N	Landsat	1986–2001	155	[2]	GLOF in 1981
Cirenmaco	86.065828E,28.067165N	Landsat	1992–2009	345	This study	–
Dig Tsho	86.586560E,27.874651N	Landsat, ALOS	2000–2007	7	[9]	GLOF on 4 Aug. 1985
Dig Tsho	86.586560E,27.874651N	Landsat	1992–2009	−2	This study	–
Galongco	85.838902E,28.321298N	Landsat	1986–2001	104	[2]	–
Galongco	85.838902E,28.321298N	Landsat, ASTER	1977–2003	117	[17]	–
Galongco	85.838902E,28.321298N	Landsat	1988–2009	116	This study	–
Gangxico	85.873906E,28.360054N	Landsat	1986–2001	118	[2]	–
Gangxico	85.873906E,28.360054N	Landsat, ASTER	1977–2003	87	[17]	–
Gangxico	85.873906E,28.360054N	Landsat	1988–2009	51	This study	–
Imja	86.922887E,27.899331N	Topographic maps, ALOS	1960s–2007	1837	[9]	–
Imja	86.922887E,27.899331N	Landsat	1992–2009	71	This study	–
Imja	86.922887E,27.899331N	Landsat, ALOS	2000–2007	11	[9]	–
Imja	86.922887E,27.899331N	ASTER	2000–2008	9	[6]	–
Imja	86.922887E,27.899331N	Landsat	2000–2009	25	This study	–
Longbasaba	88.073538E,27.948503N	Landsat	1990–2009	112	[34]	–
Longbasaba	88.073538E,27.948503N	Landsat	1989–2009	107	This study	–
Lumdin Tsho	86.613765E,27.778947N	Topographic maps, ALOS	1960s–2007	796	[9]	–
Lumdin Tsho	86.613765E,27.778947N	Landsat, ALOS	2000–2007	12	[9]	–
Lumdin Tsho	86.613765E,27.778947N	Landsat	1992–2009	52	This study	–

There are several other typical rapidly expanding glacial lakes reported by previous studies in the central Himalayas that need more attention in the future (Table 6). Galongco (Figure 4c), the largest lake among these (4.83 km^2^) in 2009, has doubled in size in the last two decades. The rates of expansion of the Galongco were also greater than 100% during the periods 1977–2003 and 1986–2001. Imja Lake (Figure 4e) and Ludin Tsho (Figure 4f) expanded by 1837% and 796%, respectively, from the 1960s to 2007 [9]. These notable expansion rates are affected by some uncertainty due to inconsistent data sources (topographic maps and ALOS). A consistent spatial resolution of the Landsat images used in this study can eliminate the effects of inconsistent data sources on glacial lake changes. Imja Lake and Ludin Tsho are still considered of potentially high risk due to their high altitudinal drops, although their areal expansion rate has decreased slightly since 1992 (Table 6). Rapid growth (by approximately 110%) has also been observed at Longbasaba Lake (Figure 4d) in the past two decades, consistent with Yao's report in 2012 [34].

### 4.2 Causes of glacial lake changes

**Figure 5:**
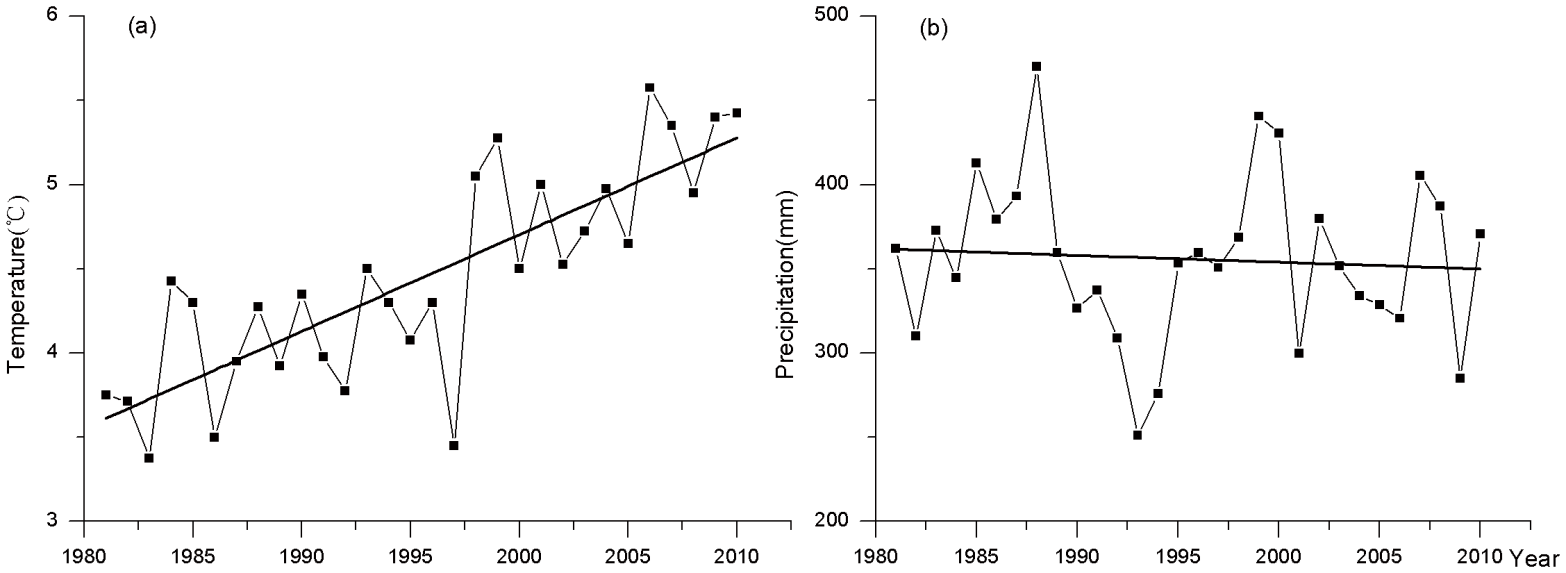
Annual mean temperature (a) and annual precipitation (b) change in the central Himalayas from 1981 to 2010.

The increasing glacier melt water is the main supply source for glacial lake expansion based on the dynamic processes of glacial lakes, glaciers and climate. Pro-glacial lakes in contact with glaciers are the greatest contributor (82.85%) of total glacial lake expansion in the study area from 1990 to 2010. The expansion of pro-glacial lakes in contact with glaciers is mainly driven by ice or glacial melt water [15]. From 1981 to 2010, the annual precipitation decreased slightly according to the four meteorological stations in the central Himalayas (Figure 5). However, the annual mean temperature increased at a rate of 0.57°C·(10a)^−1^.

An extreme warming trend that has developed in the central Himalaya has resulted in significant glacier retreat [12], [20], [35], [36]. Glacier runoff due to the accelerated glacial melting in the study area has been increasing in the past few decades [22], [37], which was the main driving force for glacial lake expansion. The impact of climate change on glacial lakes is rather complex and cannot solely account for glacial lake change [15]. The increasing light-absorbing constituents (e.g., black carbon and dust) of snow and ice [11], [38], [39] may accelerate the melting in the central Himalayan region, which is also a contributor to glacial lake expansion.

## Conclusions

The distribution and change of glacial lakes across the entire central Himalaya region for 1990, 2000, 2005 and 2010 were investigated based on Landsat images. The same spatial resolution (30 m) of Landsat images used in the four time points makes the detected glacial lake change more reliable and reduces the uncertainty resulting from using different sources of remote sensing images.

Overall, glacial lakes in the central Himalayas distinctly increased in area and number from 1990 to 2010. The larger existing glacial lakes have contributed most to the areal expansion in past decades. Most glacial lake changes occurred at the altitudinal zone between 4800 m and 5600 m at the north side and between 4500 m and 5600 m a.s.l. at the south side. The 67 glacial lakes that were selected as rapidly expanding glacial lakes imply a higher potential risk of GLOF. Among them, the Cirenmaco glacial lake has a potentially high risk to burst again after GLOF. Glacial lakes expanded on the north side of the main central Himalayas more than on the south side. The warming and increasing light-absorbing constituents of snow and ice could have accelerated the melting, which directly resulted in the glacial lake expansion.

This study gives a direct insight into what has happened to glacial lakes in the central Himalayas over the past 20 years, through remote sensing monitoring and integrated analysis. The rapidly expanded glacial lakes were selected as potential lakes at high risk for GLOF to monitor and observe continuously. To address these critical rapidly expanding glacial lakes, potential flood volume and drainage simulations for GLOFs should be carried out in the future based on in-situ observations and field measurements. Early warning systems and awareness of hazard mitigation for GLOFs are now critically needed.
