# Correction: Glacial Lake Expansion in the Central Himalayas by Landsat Images, 1990–2010

**DOI:** 10.1371/journal.pone.0092654

**Published:** 2014-03-18

**Authors:** The PLOS ONE Staff

In the Data and Methods section, there are errors in the first and second equations. Please view the correct equations here:
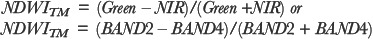
(1)

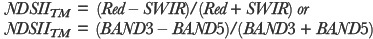
(2)

